# (9*H*-Fluoren-9-yl)methyl *N*-{(2*R*,3*R*,4*S*)-4-hy­droxy-2-[(2*S*,5*R*)-2-isopropyl-5-methyl­cyclo­hex­yloxy]-5-oxooxolan-3-yl}carbamate propan-2-ol 0.334-solvate

**DOI:** 10.1107/S1600536811055139

**Published:** 2012-01-14

**Authors:** Graeme J. Gainsford, Andreas Luxenburger

**Affiliations:** a Carbohydrate Chemistry Group, Industrial Research Limited, PO Box 31-310, Lower Hutt, New Zealand

## Abstract

The title compound, C_29_H_35_NO_6_.0.334C_3_H_8_O, a novel chiral *N*-(fluoren-9-yl­methyl­oxyxcarbon­yl) precursor, crystallizes with two independent carbamate (*M*) mol­ecules and propan-2-ol solvent mol­ecules in the unit cell. Its crystal structure has been determined from barely adequate data obtained from a multi-fragment needle crystal. In the crystal, N—H⋯O hydrogen bonds link *M* mol­ecules related by translation along the *a* axis into two independent chains. The ordered solvent mol­ecule, having a partial occupancy of 0.334, is attached to one independent *M* mol­ecule through O—H⋯O hydrogen bonds. The crystal packing exhibits weak inter­molecular C—H⋯O inter­actions and voids of 270 Å^3^ filled with randomly disordered solvent mol­ecules which were handled using the SQUEEZE methodology.

## Related literature

For details of the synthesis, see Harris *et al.* (2011 ▶). For a related structure, see: Valle *et al.* (1988 ▶). For hydrogen-bond motifs, see: Bernstein *et al.* (1995 ▶).
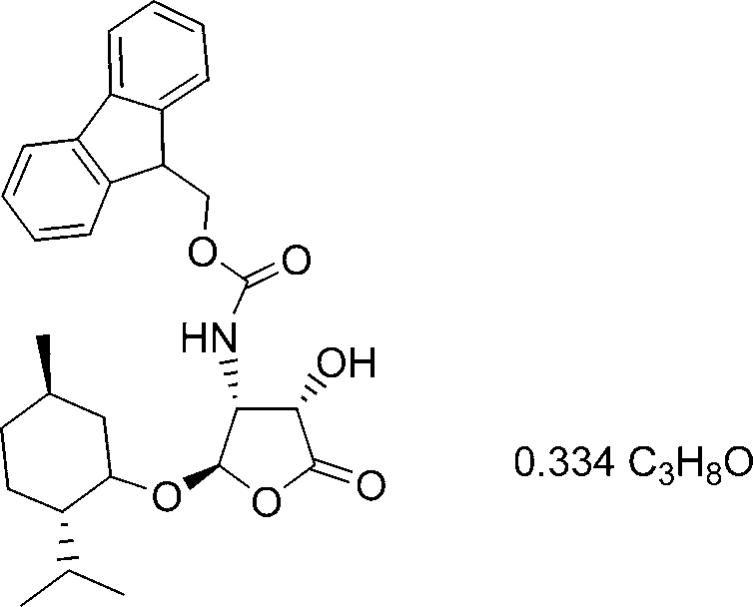


## Experimental

### 

#### Crystal data

C_29_H_35_NO_6_·0.334C_3_H_8_O
*M* *_r_* = 513.64
Triclinic, 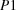

*a* = 5.1786 (2) Å
*b* = 15.3176 (5) Å
*c* = 20.3554 (14) Å
α = 98.495 (7)°
β = 92.109 (7)°
γ = 91.120 (6)°
*V* = 1595.36 (14) Å^3^
*Z* = 2
Cu *K*α radiation
μ = 0.60 mm^−1^
*T* = 123 K
0.67 × 0.10 × 0.04 mm

#### Data collection

Rigaku Spider diffractometer
Absorption correction: multi-scan (*ABSCOR*; Higashi, 1995 ▶) *T*_min_ = 0.667, *T*_max_ = 1.0
7392 measured reflections
7392 independent reflections
5189 reflections with *I* > 2σ(*I*)
*R*_int_ = 0.086
θ_max_ = 62.4°

#### Refinement

*R*[*F* ^2^ > 2σ(*F* ^2^)] = 0.095
*wR*(*F* ^2^) = 0.261
*S* = 1.00
7392 reflections
673 parameters
3 restraints
H-atom parameters constrained
Δρ_max_ = 0.41 e Å^−3^
Δρ_min_ = −0.33 e Å^−3^

### 

Data collection: *CrystalClear* (Rigaku, 2005 ▶); cell refinement: *FSProcess* in *PROCESS-AUTO* (Rigaku, 1998 ▶); data reduction: *FSProcess* in *PROCESS-AUTO*; program(s) used to solve structure: *SHELXS97* (Sheldrick, 2008 ▶); program(s) used to refine structure: *SHELXL97* (Sheldrick, 2008 ▶); molecular graphics: *ORTEP* in *WinGX* (Farrugia, 1999 ▶) and *Mercury* (Macrae *et al.*, 2008 ▶); software used to prepare material for publication: *SHELXL97*, *PLATON* (Spek, 2009 ▶) and *HYDROGEN* (Nardelli, 1999 ▶).

## Supplementary Material

Crystal structure: contains datablock(s) global, I. DOI: 10.1107/S1600536811055139/cv5219sup1.cif

Structure factors: contains datablock(s) I. DOI: 10.1107/S1600536811055139/cv5219Isup2.hkl

Supplementary material file. DOI: 10.1107/S1600536811055139/cv5219Isup3.cml

Additional supplementary materials:  crystallographic information; 3D view; checkCIF report

## Figures and Tables

**Table 1:** Hydrogen-bond geometry (Å, °)

*D*—H⋯*A*	*D*—H	H⋯*A*	*D*⋯*A*	*D*—H⋯*A*
N1—H1*N*⋯O5^i^	0.88	2.19	3.015 (9)	157
O2—H2*O*⋯O3	0.84	2.46	2.877 (9)	111
N101—H11*N*⋯O105^ii^	0.88	2.15	2.977 (10)	155
O102—H12*O*⋯N101	0.84	2.31	2.761 (10)	114
O300—H30*O*⋯O2^i^	0.86	1.95	2.707 (12)	145
C3—H3⋯O1^ii^	1.00	2.26	3.222 (10)	162
C12—H12⋯O105^ii^	0.95	2.55	3.444 (9)	158
C103—H103⋯O101^i^	1.00	2.29	3.250 (8)	161

Symmetry codes: (i) 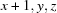; (ii) 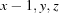.

**Fig. 1.**
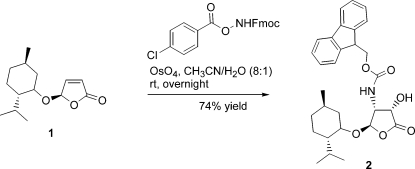
Synthesis route to title compound 2.

**Fig. 2.**
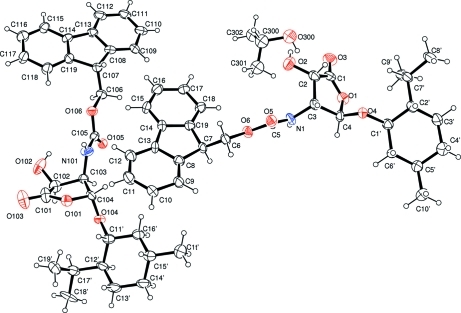
An ORTEP (Farrugia, 1999) view of 2 showing the resolved atoms with 30% probabilility ellipsoids.

**Fig. 3.**
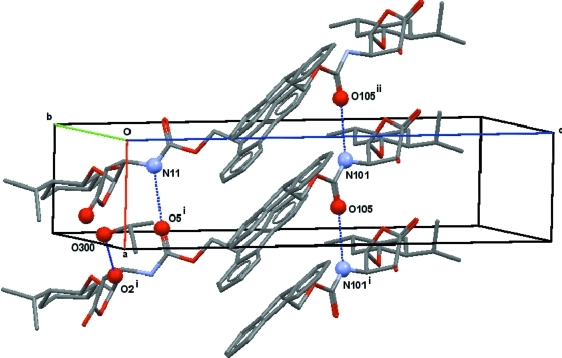
Mercury (Macrae et al., 2008) cell packing view showing the hydrogen bonds as dotted lines [symmetry codes: (i) 1 + x, y, z; (ii) x - 1, y, z].

## supplementary crystallographic information

### Comment

The title compound was prepared as part of our current research into the applicability of 4-chlorobenzoyloxycarbamates as highly efficient nitrogen reagents for the intermolecular aminohydroxylation under *base-free* reaction conditions. When the target compound, (*R*)-5-[(1*R*)-menthyloxy]-2(5*H*)-furanone 1 was treated with the Fmoc-reagent 3 (Fig. 1) using the standard aminohydroxylation conditions that we reported previously (Harris *et al.*, 2011) the title compound 2 was isolated in 74% yield. Formation of the corresponding regioisomer was not observed in our experiments.

The title compound crystallizes with two independent molecules in the asymmetric unit and one resolved partial (occupancy = 0.667) 2-propanol molecule (Fig. 2) as well as disordered 2-propanol solvent; the latter was handled using the SQUEEZE methodology (Spek, 2009), see Experimental. It seems highly likely that all the included solvent of crystallization was not stable to X-rays during the experiment, a further complicating factor which makes it impossible to define the total 2-propanol concentration in the crystal. Nevertheless, only confirmation of structure was required of this study, with the absolute configurations of C2,C102(S), C3,C103(*R*), C4,C104(*R*), C1',C11'(*R*), C2',C12'(S), C5',C15'(*R*) & C7,C107(S) expected from the synthesis. With only 61% Friedel coverage it is surprising that the chirality indications based on the oxygen anomalous dispersion is correct, although of very low statistical significance. Confidence in the structural solution and final dataset is gained from the self-consistency of the two independent molecules which are almost identical: they have an r.m.s. atom fit of 0.171 Å, r.m.s. bond fit of 0.034 Å and r.m.s. angle fit of 2.04 ° (Spek, 2009). The 5-oxotetrahydrofuran rings have envelope conformations with C3, C103 as the flap atoms respectively and the cyclohexyloxy rings are in chair conformations (Spek, 2009).

The two independent molecules form two stacks of molecules up the *a* axis (Table 1, Fig. 3) utilizing N–H···O=C hydrogen bonds with the 2-propanol bound by a O–H···O bond to one set creating a D^3^_3_(13) H bonding motif (Bernstein *et al.*, 1995). There are many *N*-(Fluoren-9-ylmethyloxyxcarbonyl) ("Fmoc") derivatives in the literature but only one *N*-(Fluoren-9-ylmethyloxyxcarbonyl)-1-aminocyclopentane-1-carboxylic acid (Valle *et al.*, 1988) attached to a 5-membered saturated ring.

### Experimental

Following the general procedure (Harris *et al.*, 2011) (*R*)-5-[(1*R*)-menthyloxy]-2(5*H*)-furanone 1 (100 mg, 0.42 mmol) was treated with osmium tetroxide (4.3 mg, 0.017 mmol) and Fmoc-reagent 3 (231.4 mg, 0.5874 mmol) at room temperature overnight.

The crude product was purified by flash column chromatography (SiO_2_, ethyl acetate/petroleum spirit 1:4 and 3:7) to yield 153 mg (74%) of 2 as a colorless foam. [α]^20^_D_ = -49 (*c* 0.545, CHCl_3_); FTIR (neat, cm^-1^) 3349, 2954, 1787, 1708, 1450, 1263, 1104, 909, 758, 740; ^1^H NMR (500 MHz, CDCl_3_) δ 7.76 (d, *J* = 7.8 Hz, 2H), 7.60–7.55 (m, 2H), 7.40 (t, *J* = 7.5 Hz, 2H), 7.31 (tt, *J* = 1.4, 7.5 Hz, 2H), 5.73 (s, 1H), 5.43 (d, *J* = 3 Hz, 1H), 4.77 (d, *J* = 5 Hz, 1H), 4.47–4.39 (m, 2H), 4.22 (d, *J* = 6.9 Hz, 1H), 4.19 (dd, *J* = 3.5, 6.5 Hz, 1H), 3.51 (td, J = 4.2, 10.7 Hz, 1H), 3.12 (*J* = 2.6 Hz, 1H), 2.20–2.09 (m, 1H), 1.95 (sept/d, *J* = 2.7, 7 Hz, 1H), 1.69–1.61 (m, 2H), 1.43–1.31 (m, 1H), 1.28–1.20 (m, 1H), 1.03–0.80 (m, 6H), 0.87 (d, *J* = 7.1 Hz, 3H), 0.74 (d, *J* = 7 Hz, 3H); ^13^C NMR (125 MHz, CDCl_3_) δ 175.82, 156.33, 143.65, 143.50, 141.33, 127.82, 127.11, 125.01, 120.05, 101.89, 78.46, 67.35, 66.60, 55.99, 47.47, 47.07, 39.48, 34.21, 31.38, 25.66, 23.02, 22.13, 20.83, 15.5; HRMS (ESI) m/*z* calcd for C_29_H_35_NO_6_Na^+^ 516.2362, obsd 516.2368. Anal. calcd for C_29_H_35_NO_6_: C, 70.57; H, 7.15; N, 2.84. Found: C, 70.39; H, 7.21; N, 2.76.

Fragile needle crystals could only be obtained by floating the 2-propanol solution onto the mounting oil.

### Refinement

All crystals mounted gave multiple crystal diffraction profiles; the largest of these was chosen. Data was then extracted by using a 30 by 30 pixel spotsize from data collected with a 5 degree scan width and redundancy 3. During processing, frames 101–127 & 408–423 were observed to be incorrectly measured with noticeable icing and so the dataset was reprocessed omitting these frames. As the compound was known to be one chiral form, space group P1 was chosen. One structural solution was achieved using *SHELXS* with the rather extreme parameter TREF 10000!

Analysis of the F_o_/F_c_ data table then showed, consistent with the observed frames, that data beyond 0.87 Å was both weak and incorrectly positioned; this data was excluded using the SHEL command. The initial solution gave a best R1 of ~18% after attempts to include partial C atoms as disordered solvent had been attempted. One 2-propanol could be identified (at about 0.7 occupancy), but the remaining solvent was fully disordered. The *PLATON* SQUEEZE processing method (Spek, 2009) was then applied with the 5756 2σ(I) data converging to an R1 of ~13%. At this point extreme F_o_>>F_c_ (at low angle) and F_o_<<F_c_ (at high angle) were noted: these were consistent with multiple crystal overlap at low angle and in adequate positioning/measurement at high angle. The 824 data with F_o_^2^/F_c_^2^ or F_c_^2^/F_o_^2^ greater than 2.0 and with the |Δ(F_o_^2^-F_c_^2^)| > 2.0 σ(F_o_^2^), which gave as R1 value of ~ 55%, were omitted. The new dataset with the 5223 2σ(I) data now converged with R1 ~10%.

The occupancy of partial resolved 2-propanol solvent was based on electron densities at the non-hydrogen atom sites determined by a refinement with fixed average isotropic U values of 0.08 e.Å^-3^. This lead to the fixed value of 0.667; the non-hydrogen atoms were then refined with one common isotropic thermal parameter.The hydrogen on the partial resolved 2-propanol oxygen was placed at its calculated position based on hydrogen bonding criteria (Nardelli, 1999).

Finally 34 individual outlier reflection were omitted to give the final convergence at R1 9.5%. There are 345 reflections missing within the final 0.87Å dataset, shell with 22 affected by backstop interactions. Although changing the relatively conservative rejection ratio criterion (of 2.0 above, for the F_o_^2^ and F_c_^2^ values) could improve further the R1 & wR2 values, it was considered that this systematic change was not justifiable.

In the absence of any significant anomalous scatterers in the molecule and the low fraction of Friedel pairs measured (0.46), the refinement of Flack parameter led to a formally inconclusive value of 0.1 (3). Therefore, in the final refinement, the Flack parameter was not refined, and the absolute configuration was assigned to correspond with that of the known chiral centres in a precursor molecule, which remained unchanged during the synthesis of the title compound.

The methyl H atoms were constrained to an ideal geometry (C—H = 0.98 Å) with *U*_iso_(H) = 1.5*U*_eq_(C), but were allowed to rotate freely about the adjacent C—C bond. All other H atoms were placed in geometrically idealized positions and constrained to ride on their parent atoms with C—H distances of 1.00 (primary), 0.99 (methylene) or 0.95 (phenyl) Å with *U*_iso_(H) = 1.2*U*_eq_(C).

### Crystal data

**Table:**

C_29_H_35_NO_6_·0.334C_3_H_8_O	*Z* = 2
*M**_r_* = 513.64	*F*(000) = 551
Triclinic, *P*1	*D*_x_ = 1.069 Mg m^−^^3^
Hall symbol: P 1	Cu *K*α radiation, λ = 1.54178 Å
*a* = 5.1786 (2) Å	Cell parameters from 11033 reflections
*b* = 15.3176 (5) Å	θ = 6.1–71.6°
*c* = 20.3554 (14) Å	µ = 0.60 mm^−^^1^
α = 98.495 (7)°	*T* = 123 K
β = 92.109 (7)°	Needle, colourless
γ = 91.120 (6)°	0.67 × 0.10 × 0.04 mm
*V* = 1595.36 (14) Å^3^	

### Data collection

**Table:**

Rigaku Spider diffractometer	7392 independent reflections
Radiation source: Rigaku MM007 rotating anode	5189 reflections with *I* > 2σ(*I*)
Rigaku VariMax-HF Confocal Optical System	*R*_int_ = 0.086
Detector resolution: 10 pixels mm^-1^	θ_max_ = 62.4°, θ_min_ = 8.9°
ω scans	*h* = −5→5
Absorption correction: multi-scan (*ABSCOR*; Higashi, 1995)	*k* = −17→17
*T*_min_ = 0.667, *T*_max_ = 1.0	*l* = −23→23
7392 measured reflections	

### Refinement

**Table:**

Refinement on *F*^2^	Secondary atom site location: difference Fourier map
Least-squares matrix: full	Hydrogen site location: inferred from neighbouring sites
*R*[*F*^2^ > 2σ(*F*^2^)] = 0.095	H-atom parameters constrained
*wR*(*F*^2^) = 0.261	*w* = 1/[σ^2^(*F*_o_^2^) + (0.1783*P*)^2^] where *P* = (*F*_o_^2^ + 2*F*_c_^2^)/3
*S* = 1.00	(Δ/σ)_max_ = 0.002
7392 reflections	Δρ_max_ = 0.41 e Å^−^^3^
673 parameters	Δρ_min_ = −0.33 e Å^−^^3^
3 restraints	Extinction correction: *SHELXL97* (Sheldrick, 2008), Fc^*^=kFc[1+0.001xFc^2^λ^3^/sin(2θ)]^-1/4^
Primary atom site location: structure-invariant direct methods	Extinction coefficient: 0.0101 (15)

### Special details

**Table:**

Geometry. All e.s.d.'s (except the e.s.d. in the dihedral angle between two l.s. planes) are estimated using the full covariance matrix. The cell e.s.d.'s are taken into account individually in the estimation of e.s.d.'s in distances, angles and torsion angles; correlations between e.s.d.'s in cell parameters are only used when they are defined by crystal symmetry. An approximate (isotropic) treatment of cell e.s.d.'s is used for estimating e.s.d.'s involving l.s. planes.
Refinement. Refinement of *F*^2^ against ALL reflections. The weighted *R*-factor *wR* and goodness of fit *S* are based on *F*^2^, conventional *R*-factors *R* are based on *F*, with *F* set to zero for negative *F*^2^. The threshold expression of *F*^2^ > σ(*F*^2^) is used only for calculating *R*-factors(gt) *etc*. and is not relevant to the choice of reflections for refinement. *R*-factors based on *F*^2^ are statistically about twice as large as those based on *F*, and *R*- factors based on ALL data will be even larger.

### Fractional atomic coordinates and isotropic or equivalent isotropic displacement parameters (Å^2^)

**Table:**

	*x*	*y*	*z*	*U*_iso_*/*U*_eq_	Occ. (<1)
O1	0.7190 (10)	0.8062 (3)	0.0823 (2)	0.0520 (12)	
O2	0.3417 (14)	0.6177 (3)	0.0835 (3)	0.0816 (19)	
H2O	0.4445	0.5858	0.0603	0.122*	
O3	0.7948 (12)	0.6754 (4)	0.0227 (3)	0.0757 (18)	
O4	0.3936 (10)	0.9052 (3)	0.0773 (2)	0.0492 (12)	
O5	−0.0682 (11)	0.7366 (3)	0.2116 (2)	0.0487 (13)	
O6	0.2298 (9)	0.6847 (3)	0.2774 (2)	0.0502 (12)	
N1	0.3549 (13)	0.7504 (4)	0.1921 (2)	0.0515 (16)	
H1N	0.5126	0.7511	0.2098	0.062*	
C1	0.6473 (16)	0.7252 (5)	0.0550 (3)	0.054 (2)	
C2	0.3736 (16)	0.7057 (5)	0.0726 (3)	0.0531 (19)	
H2	0.2594	0.7131	0.0332	0.064*	
C3	0.3102 (17)	0.7766 (4)	0.1271 (3)	0.055 (2)	
H3	0.1292	0.7965	0.1213	0.066*	
C4	0.5131 (14)	0.8518 (4)	0.1182 (3)	0.0466 (18)	
H4	0.5763	0.8859	0.1618	0.056*	
C5	0.1583 (19)	0.7259 (4)	0.2241 (3)	0.054 (2)	
C6	0.0328 (16)	0.6627 (5)	0.3177 (3)	0.056 (2)	
H6A	−0.0957	0.6227	0.2906	0.068*	
H6B	−0.0563	0.7168	0.3364	0.068*	
C7	0.1403 (14)	0.6186 (4)	0.3730 (3)	0.0408 (16)	
H7	0.2885	0.6559	0.3951	0.049*	
C8	−0.0530 (14)	0.6066 (5)	0.4256 (3)	0.0484 (18)	
C9	−0.2091 (16)	0.6674 (4)	0.4599 (3)	0.056 (2)	
H9	−0.2081	0.7269	0.4519	0.067*	
C10	−0.3727 (17)	0.6386 (5)	0.5078 (3)	0.061 (2)	
H10	−0.4769	0.6799	0.5334	0.073*	
C11	−0.3808 (18)	0.5533 (5)	0.5169 (4)	0.067 (2)	
H11	−0.4923	0.5350	0.5486	0.080*	
C12	−0.2293 (16)	0.4923 (5)	0.4809 (4)	0.058 (2)	
H12	−0.2407	0.4323	0.4874	0.070*	
C13	−0.0606 (15)	0.5169 (4)	0.4353 (3)	0.0481 (18)	
C14	0.1126 (15)	0.4682 (4)	0.3900 (3)	0.0475 (18)	
C15	0.1750 (17)	0.3796 (4)	0.3792 (4)	0.059 (2)	
H15	0.0964	0.3404	0.4050	0.071*	
C16	0.3427 (18)	0.3473 (5)	0.3334 (4)	0.063 (2)	
H16	0.3804	0.2863	0.3267	0.076*	
C17	0.4594 (17)	0.4045 (4)	0.2962 (4)	0.060 (2)	
H17	0.5745	0.3817	0.2630	0.072*	
C18	0.4115 (15)	0.4954 (4)	0.3065 (4)	0.0526 (18)	
H18	0.4995	0.5343	0.2821	0.063*	
C19	0.2358 (16)	0.5269 (4)	0.3523 (3)	0.054 (2)	
C1'	0.5544 (15)	0.9819 (4)	0.0667 (3)	0.0501 (18)	
H1'	0.7396	0.9693	0.0763	0.060*	
C2'	0.5251 (17)	0.9996 (5)	−0.0026 (3)	0.055 (2)	
H2'	0.3398	1.0135	−0.0107	0.065*	
C3'	0.686 (2)	1.0806 (5)	−0.0108 (4)	0.070 (3)	
H3'A	0.6628	1.0929	−0.0570	0.084*	
H3'B	0.8708	1.0690	−0.0027	0.084*	
C4'	0.607 (2)	1.1633 (6)	0.0382 (4)	0.074 (3)	
H4'A	0.7248	1.2138	0.0340	0.088*	
H4'B	0.4292	1.1795	0.0266	0.088*	
C5'	0.6218 (19)	1.1445 (5)	0.1094 (4)	0.065 (2)	
H5'	0.8074	1.1358	0.1214	0.078*	
C6'	0.4748 (16)	1.0615 (4)	0.1169 (3)	0.0480 (19)	
H6'A	0.2875	1.0709	0.1105	0.058*	
H6'B	0.5057	1.0482	0.1627	0.058*	
C7'	0.5880 (17)	0.9166 (5)	−0.0545 (3)	0.056 (2)	
H7'	0.4786	0.8665	−0.0437	0.068*	
C8'	0.500 (2)	0.9316 (6)	−0.1248 (4)	0.082 (3)	
H8'A	0.6208	0.9733	−0.1406	0.123*	
H8'B	0.3266	0.9558	−0.1238	0.123*	
H8'C	0.4979	0.8753	−0.1548	0.123*	
C9'	0.8581 (18)	0.8887 (6)	−0.0505 (4)	0.068 (2)	
H9'A	0.8743	0.8307	−0.0775	0.102*	
H9'B	0.9085	0.8850	−0.0041	0.102*	
H9'C	0.9711	0.9318	−0.0672	0.102*	
C10'	0.532 (2)	1.2233 (5)	0.1556 (4)	0.077 (3)	
H1'A	0.6384	1.2754	0.1506	0.115*	
H1'B	0.5496	1.2117	0.2016	0.115*	
H1'C	0.3509	1.2340	0.1447	0.115*	
O101	−0.0834 (9)	0.4245 (3)	0.6774 (2)	0.0511 (12)	
O102	0.2737 (16)	0.2349 (4)	0.6766 (4)	0.098 (2)	
H12O	0.3357	0.2209	0.6390	0.148*	
O103	−0.1716 (13)	0.3208 (5)	0.7385 (3)	0.098 (2)	
O104	0.2707 (9)	0.5213 (3)	0.6859 (2)	0.0481 (12)	
O105	0.6970 (12)	0.3018 (3)	0.5502 (2)	0.0534 (14)	
O106	0.3879 (10)	0.2247 (3)	0.4832 (2)	0.0514 (12)	
N101	0.2694 (14)	0.3225 (4)	0.5674 (3)	0.0601 (17)	
H11N	0.1117	0.3147	0.5495	0.072*	
C101	−0.0117 (19)	0.3510 (6)	0.7054 (4)	0.072 (2)	
C102	0.2559 (17)	0.3269 (5)	0.6903 (4)	0.062 (2)	
H102	0.3730	0.3499	0.7294	0.075*	
C103	0.3160 (14)	0.3765 (4)	0.6322 (3)	0.0427 (16)	
H103	0.5000	0.3984	0.6368	0.051*	
C104	0.1408 (14)	0.4531 (4)	0.6425 (3)	0.0453 (17)	
H104	0.0869	0.4728	0.5995	0.054*	
C105	0.4667 (17)	0.2855 (4)	0.5358 (3)	0.0443 (17)	
C106	0.5961 (16)	0.1848 (5)	0.4427 (3)	0.055 (2)	
H16A	0.7172	0.1552	0.4705	0.065*	
H16B	0.6937	0.2310	0.4241	0.065*	
C107	0.4784 (16)	0.1191 (4)	0.3877 (3)	0.052 (2)	
H107	0.3328	0.1471	0.3656	0.063*	
C108	0.6698 (14)	0.0873 (4)	0.3370 (3)	0.0468 (17)	
C109	0.8222 (18)	0.1341 (5)	0.3008 (3)	0.065 (2)	
H109	0.8137	0.1966	0.3046	0.077*	
C110	0.9942 (18)	0.0859 (5)	0.2573 (3)	0.065 (2)	
H110	1.1095	0.1176	0.2336	0.078*	
C111	1.0005 (17)	−0.0025 (5)	0.2481 (3)	0.063 (2)	
H111	1.1096	−0.0324	0.2161	0.076*	
C112	0.8465 (18)	−0.0507 (5)	0.2857 (3)	0.064 (2)	
H112	0.8561	−0.1132	0.2807	0.077*	
C113	0.6843 (15)	−0.0079 (4)	0.3291 (3)	0.0483 (18)	
C114	0.4974 (14)	−0.0392 (4)	0.3748 (3)	0.0446 (16)	
C115	0.4428 (17)	−0.1232 (4)	0.3870 (4)	0.058 (2)	
H115	0.5281	−0.1720	0.3637	0.070*	
C116	0.265 (2)	−0.1365 (5)	0.4329 (5)	0.083 (3)	
H116	0.2245	−0.1949	0.4404	0.099*	
C117	0.1432 (17)	−0.0657 (5)	0.4688 (4)	0.064 (2)	
H117	0.0224	−0.0757	0.5011	0.076*	
C118	0.1984 (17)	0.0199 (5)	0.4573 (4)	0.064 (2)	
H118	0.1166	0.0689	0.4814	0.077*	
C119	0.3777 (16)	0.0321 (4)	0.4093 (3)	0.055 (2)	
C11'	0.1211 (16)	0.6036 (4)	0.7002 (3)	0.0503 (18)	
H11'	−0.0677	0.5880	0.6984	0.060*	
C12'	0.2030 (17)	0.6474 (5)	0.7706 (3)	0.060 (2)	
H12'	0.3926	0.6612	0.7707	0.072*	
C13'	0.0658 (19)	0.7362 (5)	0.7842 (4)	0.071 (3)	
H3'C	−0.1227	0.7248	0.7854	0.086*	
H3'D	0.1255	0.7675	0.8284	0.086*	
C14'	0.117 (2)	0.7947 (5)	0.7322 (4)	0.077 (3)	
H4'C	0.0175	0.8493	0.7419	0.092*	
H4'D	0.3029	0.8117	0.7346	0.092*	
C15'	0.0441 (18)	0.7500 (5)	0.6631 (3)	0.061 (2)	
H15'	−0.1470	0.7384	0.6601	0.073*	
C16'	0.1762 (17)	0.6621 (5)	0.6479 (3)	0.0536 (19)	
H6'C	0.3650	0.6725	0.6465	0.064*	
H6'D	0.1139	0.6317	0.6037	0.064*	
C17'	0.1619 (19)	0.5874 (5)	0.8245 (4)	0.066 (2)	
H17'	0.2663	0.5335	0.8128	0.079*	
C18'	0.255 (2)	0.6314 (8)	0.8924 (3)	0.091 (3)	
H8D'	0.4023	0.6712	0.8882	0.137*	
H8E'	0.3096	0.5864	0.9193	0.137*	
H8F'	0.1153	0.6653	0.9141	0.137*	
C19'	−0.1191 (18)	0.5573 (6)	0.8270 (4)	0.070 (2)	
H9D'	−0.1286	0.5067	0.8512	0.105*	
H9E'	−0.1912	0.5401	0.7816	0.105*	
H9F'	−0.2185	0.6057	0.8497	0.105*	
C1T'	0.112 (2)	0.8087 (6)	0.6105 (5)	0.092 (3)	
H1'D	0.0146	0.8632	0.6181	0.137*	
H1'E	0.0663	0.7770	0.5660	0.137*	
H1'F	0.2975	0.8230	0.6140	0.137*	
O300	0.945 (2)	0.5053 (6)	0.0366 (5)	0.0845 (17)*	0.667
H30O	1.0155	0.5544	0.0562	0.127*	0.667
C300	0.859 (3)	0.4525 (10)	0.0876 (7)	0.0845 (17)*	0.667
H300	0.6951	0.4184	0.0735	0.127*	0.667
C302	1.088 (3)	0.3928 (10)	0.0916 (7)	0.0845 (17)*	0.667
H32A	1.1029	0.3545	0.0490	0.127*	0.667
H32B	1.0632	0.3564	0.1268	0.127*	0.667
H32C	1.2472	0.4289	0.1018	0.127*	0.667
C301	0.841 (3)	0.5067 (10)	0.1489 (7)	0.0845 (17)*	0.667
H31A	0.9897	0.4972	0.1781	0.127*	0.667
H31B	0.6811	0.4922	0.1695	0.127*	0.667
H31C	0.8408	0.5687	0.1420	0.127*	0.667

### Atomic displacement parameters (Å^2^)

**Table:**

	*U*^11^	*U*^22^	*U*^33^	*U*^12^	*U*^13^	*U*^23^
O1	0.041 (3)	0.064 (3)	0.054 (3)	0.017 (2)	0.008 (2)	0.014 (2)
O2	0.108 (6)	0.051 (3)	0.088 (4)	−0.001 (3)	0.020 (4)	0.012 (3)
O3	0.082 (5)	0.079 (4)	0.066 (3)	0.023 (3)	0.018 (3)	0.001 (3)
O4	0.064 (4)	0.042 (2)	0.043 (2)	0.001 (2)	0.005 (2)	0.0110 (18)
O5	0.054 (4)	0.045 (2)	0.049 (3)	0.011 (2)	0.007 (2)	0.0116 (19)
O6	0.047 (3)	0.056 (3)	0.049 (2)	0.001 (2)	0.015 (2)	0.012 (2)
N1	0.062 (5)	0.063 (3)	0.034 (3)	0.012 (3)	0.010 (3)	0.019 (2)
C1	0.065 (6)	0.064 (5)	0.031 (3)	0.007 (4)	0.007 (3)	−0.001 (3)
C2	0.053 (6)	0.065 (4)	0.041 (3)	−0.002 (4)	0.010 (3)	0.005 (3)
C3	0.080 (6)	0.045 (3)	0.045 (3)	0.020 (4)	0.019 (4)	0.015 (3)
C4	0.055 (5)	0.041 (3)	0.048 (3)	0.002 (3)	0.018 (3)	0.016 (3)
C5	0.085 (7)	0.050 (4)	0.031 (3)	0.017 (4)	0.023 (4)	0.016 (3)
C6	0.080 (6)	0.052 (4)	0.042 (3)	0.015 (4)	0.020 (4)	0.017 (3)
C7	0.038 (5)	0.045 (3)	0.042 (3)	−0.001 (3)	0.015 (3)	0.012 (3)
C8	0.043 (5)	0.056 (4)	0.045 (3)	−0.004 (3)	0.007 (3)	0.005 (3)
C9	0.074 (6)	0.050 (4)	0.048 (4)	0.013 (4)	0.021 (4)	0.011 (3)
C10	0.076 (7)	0.065 (5)	0.042 (3)	−0.004 (4)	0.013 (4)	0.009 (3)
C11	0.080 (7)	0.078 (5)	0.047 (4)	0.009 (5)	0.008 (4)	0.021 (4)
C12	0.053 (6)	0.069 (5)	0.053 (4)	−0.005 (4)	0.007 (4)	0.013 (3)
C13	0.057 (5)	0.043 (3)	0.048 (3)	−0.009 (3)	0.010 (3)	0.021 (3)
C14	0.049 (5)	0.048 (4)	0.049 (4)	−0.004 (3)	0.005 (3)	0.019 (3)
C15	0.079 (7)	0.039 (3)	0.062 (4)	−0.008 (4)	0.002 (4)	0.020 (3)
C16	0.071 (6)	0.045 (4)	0.072 (5)	0.001 (4)	0.005 (4)	0.005 (4)
C17	0.080 (7)	0.043 (4)	0.056 (4)	0.009 (4)	0.015 (4)	−0.003 (3)
C18	0.041 (5)	0.050 (4)	0.065 (4)	−0.009 (3)	0.011 (4)	0.003 (3)
C19	0.074 (6)	0.044 (4)	0.045 (3)	0.011 (4)	0.013 (4)	0.005 (3)
C1'	0.049 (5)	0.056 (4)	0.046 (4)	−0.003 (4)	0.004 (3)	0.007 (3)
C2'	0.066 (6)	0.059 (4)	0.041 (4)	0.002 (4)	0.020 (4)	0.010 (3)
C3'	0.090 (8)	0.072 (5)	0.051 (4)	−0.016 (5)	0.011 (4)	0.016 (4)
C4'	0.086 (8)	0.067 (5)	0.069 (5)	−0.005 (5)	0.006 (5)	0.015 (4)
C5'	0.088 (7)	0.051 (4)	0.057 (4)	0.011 (4)	0.009 (4)	0.012 (3)
C6'	0.072 (6)	0.035 (3)	0.037 (3)	0.008 (3)	0.009 (3)	0.003 (2)
C7'	0.066 (6)	0.069 (4)	0.033 (3)	−0.015 (4)	0.005 (3)	0.005 (3)
C8'	0.104 (8)	0.103 (6)	0.035 (4)	−0.030 (6)	0.009 (4)	0.002 (4)
C9'	0.059 (7)	0.083 (6)	0.057 (4)	−0.002 (5)	0.018 (4)	−0.010 (4)
C10'	0.105 (8)	0.051 (4)	0.074 (5)	0.006 (5)	0.003 (5)	0.005 (4)
O101	0.031 (3)	0.062 (3)	0.058 (3)	0.002 (2)	0.010 (2)	0.004 (2)
O102	0.112 (6)	0.046 (3)	0.142 (6)	−0.004 (3)	−0.002 (5)	0.031 (4)
O103	0.071 (5)	0.143 (6)	0.093 (4)	0.000 (4)	0.014 (4)	0.063 (4)
O104	0.053 (3)	0.045 (2)	0.044 (2)	0.007 (2)	0.003 (2)	−0.0040 (19)
O105	0.071 (4)	0.039 (2)	0.052 (3)	0.006 (3)	0.015 (3)	0.0076 (19)
O106	0.054 (4)	0.047 (2)	0.050 (2)	0.001 (2)	0.006 (2)	−0.002 (2)
N101	0.049 (5)	0.061 (4)	0.064 (4)	−0.005 (3)	0.006 (3)	−0.009 (3)
C101	0.061 (7)	0.090 (6)	0.071 (5)	−0.009 (5)	−0.001 (5)	0.039 (5)
C102	0.061 (6)	0.062 (5)	0.064 (5)	0.012 (4)	−0.008 (4)	0.015 (4)
C103	0.035 (5)	0.048 (3)	0.044 (3)	−0.003 (3)	0.008 (3)	0.002 (3)
C104	0.049 (5)	0.055 (4)	0.032 (3)	0.002 (3)	0.012 (3)	0.005 (3)
C105	0.044 (6)	0.039 (3)	0.049 (4)	−0.006 (4)	0.003 (4)	0.006 (3)
C106	0.063 (6)	0.052 (4)	0.047 (4)	0.005 (4)	0.018 (4)	−0.002 (3)
C107	0.074 (6)	0.040 (3)	0.042 (3)	0.004 (4)	0.011 (4)	0.002 (3)
C108	0.044 (5)	0.051 (4)	0.041 (3)	0.005 (3)	−0.002 (3)	−0.007 (3)
C109	0.097 (8)	0.057 (4)	0.040 (3)	−0.004 (4)	0.024 (4)	0.001 (3)
C110	0.080 (7)	0.066 (5)	0.043 (4)	0.007 (4)	−0.001 (4)	−0.009 (3)
C111	0.078 (7)	0.064 (5)	0.044 (4)	0.018 (4)	0.010 (4)	−0.007 (3)
C112	0.080 (7)	0.061 (5)	0.048 (4)	0.017 (4)	0.008 (4)	−0.009 (3)
C113	0.045 (5)	0.047 (4)	0.049 (4)	0.006 (3)	0.009 (3)	−0.004 (3)
C114	0.037 (5)	0.038 (3)	0.057 (4)	0.004 (3)	0.000 (3)	−0.001 (3)
C115	0.073 (6)	0.039 (4)	0.064 (4)	0.006 (4)	0.001 (4)	0.008 (3)
C116	0.127 (9)	0.039 (4)	0.083 (5)	0.017 (5)	0.033 (6)	0.003 (4)
C117	0.064 (6)	0.044 (4)	0.084 (5)	−0.002 (4)	0.016 (4)	0.014 (4)
C118	0.068 (7)	0.046 (4)	0.079 (5)	0.005 (4)	0.028 (4)	0.004 (3)
C119	0.078 (6)	0.034 (3)	0.054 (4)	0.011 (3)	0.013 (4)	0.010 (3)
C11'	0.046 (5)	0.049 (4)	0.054 (4)	0.014 (3)	0.007 (3)	−0.003 (3)
C12'	0.067 (6)	0.072 (5)	0.038 (3)	0.001 (4)	0.019 (4)	−0.008 (3)
C13'	0.086 (7)	0.064 (5)	0.060 (4)	0.024 (4)	0.023 (5)	−0.014 (4)
C14'	0.118 (9)	0.056 (4)	0.053 (4)	0.025 (5)	0.008 (5)	−0.012 (3)
C15'	0.071 (7)	0.056 (4)	0.051 (4)	0.000 (4)	0.001 (4)	−0.003 (3)
C16'	0.055 (5)	0.059 (4)	0.044 (4)	0.003 (4)	−0.009 (3)	0.002 (3)
C17'	0.083 (7)	0.065 (4)	0.047 (4)	0.016 (4)	0.008 (4)	−0.003 (3)
C18'	0.094 (8)	0.144 (9)	0.029 (3)	0.044 (6)	0.014 (4)	−0.016 (4)
C19'	0.064 (7)	0.089 (6)	0.058 (5)	0.002 (5)	0.018 (4)	0.012 (4)
C1T'	0.135 (10)	0.064 (5)	0.080 (6)	0.010 (6)	0.010 (6)	0.019 (4)

### Geometric parameters (Å, °)

**Table:**

O1—C1	1.321 (9)	O104—C11'	1.489 (7)
O1—C4	1.448 (8)	O105—C105	1.230 (8)
O2—C2	1.406 (9)	O106—C105	1.357 (8)
O2—H2O	0.8400	O106—C106	1.468 (8)
O3—C1	1.229 (8)	N101—C105	1.320 (9)
O4—C4	1.389 (8)	N101—C103	1.460 (9)
O4—C1'	1.473 (8)	N101—H11N	0.8800
O5—C5	1.211 (10)	C101—C102	1.473 (12)
O6—C5	1.377 (8)	C102—C103	1.535 (10)
O6—C6	1.399 (8)	C102—H102	1.0000
N1—C5	1.307 (9)	C103—C104	1.492 (9)
N1—C3	1.451 (8)	C103—H103	1.0000
N1—H1N	0.8800	C104—H104	1.0000
C1—C2	1.509 (11)	C106—C107	1.493 (10)
C2—C3	1.483 (9)	C106—H16A	0.9900
C2—H2	1.0000	C106—H16B	0.9900
C3—C4	1.579 (10)	C107—C108	1.494 (9)
C3—H3	1.0000	C107—C119	1.550 (9)
C4—H4	1.0000	C107—H107	1.0000
C6—C7	1.492 (9)	C108—C109	1.363 (10)
C6—H6A	0.9900	C108—C113	1.447 (9)
C6—H6B	0.9900	C109—C110	1.420 (10)
C7—C19	1.504 (8)	C109—H109	0.9500
C7—C8	1.521 (8)	C110—C111	1.340 (11)
C7—H7	1.0000	C110—H110	0.9500
C8—C9	1.373 (9)	C111—C112	1.398 (11)
C8—C13	1.417 (9)	C111—H111	0.9500
C9—C10	1.427 (9)	C112—C113	1.350 (9)
C9—H9	0.9500	C112—H112	0.9500
C10—C11	1.346 (10)	C113—C114	1.487 (9)
C10—H10	0.9500	C114—C115	1.371 (9)
C11—C12	1.376 (11)	C114—C119	1.380 (9)
C11—H11	0.9500	C115—C116	1.369 (11)
C12—C13	1.383 (9)	C115—H115	0.9500
C12—H12	0.9500	C116—C117	1.391 (10)
C13—C14	1.447 (9)	C116—H116	0.9500
C14—C15	1.389 (9)	C117—C118	1.392 (10)
C14—C19	1.422 (9)	C117—H117	0.9500
C15—C16	1.344 (10)	C118—C119	1.403 (10)
C15—H15	0.9500	C118—H118	0.9500
C16—C17	1.383 (11)	C11'—C16'	1.521 (10)
C16—H16	0.9500	C11'—C12'	1.531 (10)
C17—C18	1.406 (9)	C11'—H11'	1.0000
C17—H17	0.9500	C12'—C13'	1.539 (10)
C18—C19	1.370 (9)	C12'—C17'	1.549 (10)
C18—H18	0.9500	C12'—H12'	1.0000
C1'—C2'	1.479 (9)	C13'—C14'	1.512 (11)
C1'—C6'	1.544 (8)	C13'—H3'C	0.9900
C1'—H1'	1.0000	C13'—H3'D	0.9900
C2'—C3'	1.514 (11)	C14'—C15'	1.503 (11)
C2'—C7'	1.575 (10)	C14'—H4'C	0.9900
C2'—H2'	1.0000	C14'—H4'D	0.9900
C3'—C4'	1.562 (12)	C15'—C16'	1.518 (10)
C3'—H3'A	0.9900	C15'—C1T'	1.541 (11)
C3'—H3'B	0.9900	C15'—H15'	1.0000
C4'—C5'	1.516 (11)	C16'—H6'C	0.9900
C4'—H4'A	0.9900	C16'—H6'D	0.9900
C4'—H4'B	0.9900	C17'—C18'	1.504 (12)
C5'—C6'	1.499 (10)	C17'—C19'	1.523 (13)
C5'—C10'	1.508 (10)	C17'—H17'	1.0000
C5'—H5'	1.0000	C18'—H8D'	0.9800
C6'—H6'A	0.9900	C18'—H8E'	0.9800
C6'—H6'B	0.9900	C18'—H8F'	0.9800
C7'—C9'	1.473 (12)	C19'—H9D'	0.9800
C7'—C8'	1.536 (10)	C19'—H9E'	0.9800
C7'—H7'	1.0000	C19'—H9F'	0.9800
C8'—H8'A	0.9800	C1T'—H1'D	0.9800
C8'—H8'B	0.9800	C1T'—H1'E	0.9800
C8'—H8'C	0.9800	C1T'—H1'F	0.9800
C9'—H9'A	0.9800	O300—C300	1.483 (17)
C9'—H9'B	0.9800	O300—H30O	0.864 (10)
C9'—H9'C	0.9800	C300—C301	1.400 (19)
C10'—H1'A	0.9800	C300—C302	1.52 (2)
C10'—H1'B	0.9800	C300—H300	1.0000
C10'—H1'C	0.9800	C302—H32A	0.9800
O101—C101	1.386 (9)	C302—H32B	0.9800
O101—C104	1.476 (8)	C302—H32C	0.9800
O102—C102	1.401 (9)	C301—H31A	0.9800
O102—H12O	0.8400	C301—H31B	0.9800
O103—C101	1.213 (10)	C301—H31C	0.9800
O104—C104	1.407 (8)		
			
C1—O1—C4	112.0 (6)	O102—C102—C101	110.1 (8)
C2—O2—H2O	109.5	O102—C102—C103	115.2 (6)
C4—O4—C1'	113.5 (5)	C101—C102—C103	103.6 (6)
C5—O6—C6	117.0 (6)	O102—C102—H102	109.3
C5—N1—C3	119.3 (7)	C101—C102—H102	109.3
C5—N1—H1N	120.3	C103—C102—H102	109.3
C3—N1—H1N	120.3	N101—C103—C104	112.3 (6)
O3—C1—O1	122.1 (7)	N101—C103—C102	112.9 (6)
O3—C1—C2	127.9 (7)	C104—C103—C102	102.8 (5)
O1—C1—C2	109.9 (6)	N101—C103—H103	109.6
O2—C2—C3	117.8 (6)	C104—C103—H103	109.6
O2—C2—C1	111.4 (6)	C102—C103—H103	109.6
C3—C2—C1	106.0 (6)	O104—C104—O101	107.2 (4)
O2—C2—H2	107.0	O104—C104—C103	107.8 (6)
C3—C2—H2	107.0	O101—C104—C103	106.6 (5)
C1—C2—H2	107.0	O104—C104—H104	111.6
N1—C3—C2	112.2 (5)	O101—C104—H104	111.6
N1—C3—C4	108.8 (7)	C103—C104—H104	111.6
C2—C3—C4	102.0 (5)	O105—C105—N101	126.3 (7)
N1—C3—H3	111.2	O105—C105—O106	121.8 (6)
C2—C3—H3	111.2	N101—C105—O106	111.8 (7)
C4—C3—H3	111.2	O106—C106—C107	108.5 (6)
O4—C4—O1	108.2 (5)	O106—C106—H16A	110.0
O4—C4—C3	106.7 (6)	C107—C106—H16A	110.0
O1—C4—C3	104.6 (5)	O106—C106—H16B	110.0
O4—C4—H4	112.3	C107—C106—H16B	110.0
O1—C4—H4	112.3	H16A—C106—H16B	108.4
C3—C4—H4	112.3	C106—C107—C108	112.1 (7)
O5—C5—N1	126.9 (6)	C106—C107—C119	114.9 (5)
O5—C5—O6	119.8 (6)	C108—C107—C119	102.6 (5)
N1—C5—O6	113.3 (8)	C106—C107—H107	109.0
O6—C6—C7	110.6 (6)	C108—C107—H107	109.0
O6—C6—H6A	109.5	C119—C107—H107	109.0
C7—C6—H6A	109.5	C109—C108—C113	119.9 (6)
O6—C6—H6B	109.5	C109—C108—C107	129.7 (6)
C7—C6—H6B	109.5	C113—C108—C107	110.5 (6)
H6A—C6—H6B	108.1	C108—C109—C110	117.3 (7)
C6—C7—C19	115.1 (5)	C108—C109—H109	121.3
C6—C7—C8	113.8 (6)	C110—C109—H109	121.3
C19—C7—C8	103.6 (5)	C111—C110—C109	122.7 (8)
C6—C7—H7	108.0	C111—C110—H110	118.7
C19—C7—H7	108.0	C109—C110—H110	118.7
C8—C7—H7	108.0	C110—C111—C112	120.0 (7)
C9—C8—C13	121.2 (6)	C110—C111—H111	120.0
C9—C8—C7	129.6 (6)	C112—C111—H111	120.0
C13—C8—C7	109.2 (5)	C113—C112—C111	119.6 (7)
C8—C9—C10	118.2 (6)	C113—C112—H112	120.2
C8—C9—H9	120.9	C111—C112—H112	120.2
C10—C9—H9	120.9	C112—C113—C108	120.4 (7)
C11—C10—C9	120.6 (7)	C112—C113—C114	132.5 (6)
C11—C10—H10	119.7	C108—C113—C114	107.1 (5)
C9—C10—H10	119.7	C115—C114—C119	120.5 (6)
C10—C11—C12	120.7 (7)	C115—C114—C113	129.9 (6)
C10—C11—H11	119.6	C119—C114—C113	109.6 (5)
C12—C11—H11	119.6	C116—C115—C114	119.8 (7)
C11—C12—C13	121.2 (7)	C116—C115—H115	120.1
C11—C12—H12	119.4	C114—C115—H115	120.1
C13—C12—H12	119.4	C115—C116—C117	120.9 (7)
C12—C13—C8	118.0 (6)	C115—C116—H116	119.6
C12—C13—C14	133.3 (6)	C117—C116—H116	119.6
C8—C13—C14	108.7 (5)	C116—C117—C118	119.9 (7)
C15—C14—C19	118.5 (6)	C116—C117—H117	120.1
C15—C14—C13	131.9 (6)	C118—C117—H117	120.1
C19—C14—C13	109.6 (5)	C117—C118—C119	118.4 (7)
C16—C15—C14	122.4 (6)	C117—C118—H118	120.8
C16—C15—H15	118.8	C119—C118—H118	120.8
C14—C15—H15	118.8	C114—C119—C118	120.5 (6)
C15—C16—C17	118.9 (6)	C114—C119—C107	110.3 (6)
C15—C16—H16	120.5	C118—C119—C107	129.2 (6)
C17—C16—H16	120.5	O104—C11'—C16'	108.8 (5)
C16—C17—C18	121.5 (7)	O104—C11'—C12'	107.3 (6)
C16—C17—H17	119.3	C16'—C11'—C12'	112.6 (6)
C18—C17—H17	119.3	O104—C11'—H11'	109.4
C19—C18—C17	118.9 (7)	C16'—C11'—H11'	109.4
C19—C18—H18	120.6	C12'—C11'—H11'	109.4
C17—C18—H18	120.6	C11'—C12'—C13'	107.9 (7)
C18—C19—C14	119.8 (6)	C11'—C12'—C17'	113.7 (6)
C18—C19—C7	131.2 (6)	C13'—C12'—C17'	113.6 (6)
C14—C19—C7	108.9 (5)	C11'—C12'—H12'	107.1
O4—C1'—C2'	111.2 (6)	C13'—C12'—H12'	107.1
O4—C1'—C6'	107.6 (5)	C17'—C12'—H12'	107.1
C2'—C1'—C6'	111.8 (5)	C14'—C13'—C12'	112.4 (6)
O4—C1'—H1'	108.7	C14'—C13'—H3'C	109.1
C2'—C1'—H1'	108.7	C12'—C13'—H3'C	109.1
C6'—C1'—H1'	108.7	C14'—C13'—H3'D	109.1
C1'—C2'—C3'	109.6 (7)	C12'—C13'—H3'D	109.1
C1'—C2'—C7'	112.2 (6)	H3'C—C13'—H3'D	107.9
C3'—C2'—C7'	112.7 (6)	C15'—C14'—C13'	112.4 (7)
C1'—C2'—H2'	107.3	C15'—C14'—H4'C	109.1
C3'—C2'—H2'	107.3	C13'—C14'—H4'C	109.1
C7'—C2'—H2'	107.3	C15'—C14'—H4'D	109.1
C2'—C3'—C4'	111.7 (6)	C13'—C14'—H4'D	109.1
C2'—C3'—H3'A	109.3	H4'C—C14'—H4'D	107.9
C4'—C3'—H3'A	109.3	C14'—C15'—C16'	111.0 (7)
C2'—C3'—H3'B	109.3	C14'—C15'—C1T'	111.6 (7)
C4'—C3'—H3'B	109.3	C16'—C15'—C1T'	109.6 (6)
H3'A—C3'—H3'B	107.9	C14'—C15'—H15'	108.2
C5'—C4'—C3'	110.7 (7)	C16'—C15'—H15'	108.2
C5'—C4'—H4'A	109.5	C1T'—C15'—H15'	108.2
C3'—C4'—H4'A	109.5	C15'—C16'—C11'	111.0 (6)
C5'—C4'—H4'B	109.5	C15'—C16'—H6'C	109.4
C3'—C4'—H4'B	109.5	C11'—C16'—H6'C	109.4
H4'A—C4'—H4'B	108.1	C15'—C16'—H6'D	109.4
C6'—C5'—C10'	112.1 (6)	C11'—C16'—H6'D	109.4
C6'—C5'—C4'	111.9 (7)	H6'C—C16'—H6'D	108.0
C10'—C5'—C4'	110.0 (6)	C18'—C17'—C19'	109.4 (7)
C6'—C5'—H5'	107.5	C18'—C17'—C12'	112.1 (8)
C10'—C5'—H5'	107.5	C19'—C17'—C12'	112.5 (7)
C4'—C5'—H5'	107.5	C18'—C17'—H17'	107.6
C5'—C6'—C1'	112.2 (5)	C19'—C17'—H17'	107.6
C5'—C6'—H6'A	109.2	C12'—C17'—H17'	107.6
C1'—C6'—H6'A	109.2	C17'—C18'—H8D'	109.5
C5'—C6'—H6'B	109.2	C17'—C18'—H8E'	109.5
C1'—C6'—H6'B	109.2	H8D'—C18'—H8E'	109.5
H6'A—C6'—H6'B	107.9	C17'—C18'—H8F'	109.5
C9'—C7'—C8'	112.8 (7)	H8D'—C18'—H8F'	109.5
C9'—C7'—C2'	114.2 (7)	H8E'—C18'—H8F'	109.5
C8'—C7'—C2'	109.9 (7)	C17'—C19'—H9D'	109.5
C9'—C7'—H7'	106.4	C17'—C19'—H9E'	109.5
C8'—C7'—H7'	106.4	H9D'—C19'—H9E'	109.5
C2'—C7'—H7'	106.4	C17'—C19'—H9F'	109.5
C7'—C8'—H8'A	109.5	H9D'—C19'—H9F'	109.5
C7'—C8'—H8'B	109.5	H9E'—C19'—H9F'	109.5
H8'A—C8'—H8'B	109.5	C15'—C1T'—H1'D	109.5
C7'—C8'—H8'C	109.5	C15'—C1T'—H1'E	109.5
H8'A—C8'—H8'C	109.5	H1'D—C1T'—H1'E	109.5
H8'B—C8'—H8'C	109.5	C15'—C1T'—H1'F	109.5
C7'—C9'—H9'A	109.5	H1'D—C1T'—H1'F	109.5
C7'—C9'—H9'B	109.5	H1'E—C1T'—H1'F	109.5
H9'A—C9'—H9'B	109.5	C300—O300—H30O	109.0 (10)
C7'—C9'—H9'C	109.5	C301—C300—O300	110.2 (12)
H9'A—C9'—H9'C	109.5	C301—C300—C302	108.8 (14)
H9'B—C9'—H9'C	109.5	O300—C300—C302	100.6 (11)
C5'—C10'—H1'A	109.5	C301—C300—H300	112.2
C5'—C10'—H1'B	109.5	O300—C300—H300	112.2
H1'A—C10'—H1'B	109.5	C302—C300—H300	112.2
C5'—C10'—H1'C	109.5	C300—C302—H32A	109.5
H1'A—C10'—H1'C	109.5	C300—C302—H32B	109.5
H1'B—C10'—H1'C	109.5	H32A—C302—H32B	109.5
C101—O101—C104	107.5 (6)	C300—C302—H32C	109.5
C102—O102—H12O	109.5	H32A—C302—H32C	109.5
C104—O104—C11'	114.3 (5)	H32B—C302—H32C	109.5
C105—O106—C106	115.1 (6)	C300—C301—H31A	109.5
C105—N101—C103	119.2 (7)	C300—C301—H31B	109.5
C105—N101—H11N	120.4	H31A—C301—H31B	109.5
C103—N101—H11N	120.4	C300—C301—H31C	109.5
O103—C101—O101	116.2 (8)	H31A—C301—H31C	109.5
O103—C101—C102	132.4 (8)	H31B—C301—H31C	109.5
O101—C101—C102	111.3 (6)		
			
C4—O1—C1—O3	−179.5 (6)	C104—O101—C101—O103	177.1 (7)
C4—O1—C1—C2	−1.9 (7)	C104—O101—C101—C102	−0.7 (9)
O3—C1—C2—O2	34.6 (10)	O103—C101—C102—O102	42.4 (14)
O1—C1—C2—O2	−142.8 (6)	O101—C101—C102—O102	−140.2 (7)
O3—C1—C2—C3	163.9 (7)	O103—C101—C102—C103	166.0 (10)
O1—C1—C2—C3	−13.5 (8)	O101—C101—C102—C103	−16.6 (10)
C5—N1—C3—C2	−101.7 (8)	C105—N101—C103—C104	144.6 (6)
C5—N1—C3—C4	146.2 (6)	C105—N101—C103—C102	−99.7 (8)
O2—C2—C3—N1	30.4 (11)	O102—C102—C103—N101	25.6 (10)
C1—C2—C3—N1	−95.1 (7)	C101—C102—C103—N101	−94.7 (7)
O2—C2—C3—C4	146.7 (7)	O102—C102—C103—C104	146.8 (7)
C1—C2—C3—C4	21.2 (7)	C101—C102—C103—C104	26.5 (8)
C1'—O4—C4—O1	−70.8 (7)	C11'—O104—C104—O101	−66.5 (6)
C1'—O4—C4—C3	177.1 (5)	C11'—O104—C104—C103	179.0 (5)
C1—O1—C4—O4	−98.0 (6)	C101—O101—C104—O104	−96.8 (7)
C1—O1—C4—C3	15.5 (7)	C101—O101—C104—C103	18.5 (7)
N1—C3—C4—O4	−149.1 (5)	N101—C103—C104—O104	−151.2 (5)
C2—C3—C4—O4	92.2 (6)	C102—C103—C104—O104	87.1 (6)
N1—C3—C4—O1	96.4 (6)	N101—C103—C104—O101	93.9 (6)
C2—C3—C4—O1	−22.3 (7)	C102—C103—C104—O101	−27.7 (7)
C3—N1—C5—O5	−14.1 (10)	C103—N101—C105—O105	−11.9 (10)
C3—N1—C5—O6	166.4 (6)	C103—N101—C105—O106	168.3 (5)
C6—O6—C5—O5	−4.6 (9)	C106—O106—C105—O105	−3.8 (8)
C6—O6—C5—N1	175.0 (6)	C106—O106—C105—N101	176.0 (5)
C5—O6—C6—C7	179.9 (6)	C105—O106—C106—C107	179.4 (5)
O6—C6—C7—C19	−70.4 (8)	O106—C106—C107—C108	169.6 (5)
O6—C6—C7—C8	170.2 (5)	O106—C106—C107—C119	−73.8 (8)
C6—C7—C8—C9	−51.1 (10)	C106—C107—C108—C109	−55.7 (10)
C19—C7—C8—C9	−176.8 (8)	C119—C107—C108—C109	−179.5 (8)
C6—C7—C8—C13	127.4 (7)	C106—C107—C108—C113	123.5 (7)
C19—C7—C8—C13	1.7 (8)	C119—C107—C108—C113	−0.3 (8)
C13—C8—C9—C10	2.4 (11)	C113—C108—C109—C110	−1.1 (11)
C7—C8—C9—C10	−179.2 (7)	C107—C108—C109—C110	178.0 (8)
C8—C9—C10—C11	−2.7 (12)	C108—C109—C110—C111	3.8 (12)
C9—C10—C11—C12	0.8 (13)	C109—C110—C111—C112	−4.6 (13)
C10—C11—C12—C13	1.4 (13)	C110—C111—C112—C113	2.7 (12)
C11—C12—C13—C8	−1.6 (12)	C111—C112—C113—C108	−0.2 (12)
C11—C12—C13—C14	−177.9 (8)	C111—C112—C113—C114	179.2 (8)
C9—C8—C13—C12	−0.4 (11)	C109—C108—C113—C112	−0.5 (11)
C7—C8—C13—C12	−179.0 (7)	C107—C108—C113—C112	−179.8 (7)
C9—C8—C13—C14	176.8 (7)	C109—C108—C113—C114	179.9 (7)
C7—C8—C13—C14	−1.9 (8)	C107—C108—C113—C114	0.6 (8)
C12—C13—C14—C15	−3.0 (15)	C112—C113—C114—C115	2.2 (14)
C8—C13—C14—C15	−179.6 (8)	C108—C113—C114—C115	−178.3 (8)
C12—C13—C14—C19	177.8 (8)	C112—C113—C114—C119	179.8 (8)
C8—C13—C14—C19	1.3 (9)	C108—C113—C114—C119	−0.7 (8)
C19—C14—C15—C16	−1.5 (12)	C119—C114—C115—C116	1.1 (12)
C13—C14—C15—C16	179.5 (8)	C113—C114—C115—C116	178.5 (8)
C14—C15—C16—C17	0.7 (13)	C114—C115—C116—C117	−1.7 (15)
C15—C16—C17—C18	1.7 (12)	C115—C116—C117—C118	1.1 (15)
C16—C17—C18—C19	−3.1 (12)	C116—C117—C118—C119	0.0 (14)
C17—C18—C19—C14	2.2 (12)	C115—C114—C119—C118	0.0 (12)
C17—C18—C19—C7	−178.5 (8)	C113—C114—C119—C118	−177.9 (7)
C15—C14—C19—C18	0.0 (12)	C115—C114—C119—C107	178.4 (7)
C13—C14—C19—C18	179.2 (7)	C113—C114—C119—C107	0.5 (9)
C15—C14—C19—C7	−179.5 (7)	C117—C118—C119—C114	−0.6 (13)
C13—C14—C19—C7	−0.2 (9)	C117—C118—C119—C107	−178.6 (8)
C6—C7—C19—C18	54.9 (12)	C106—C107—C119—C114	−122.1 (7)
C8—C7—C19—C18	179.8 (8)	C108—C107—C119—C114	−0.1 (8)
C6—C7—C19—C14	−125.8 (7)	C106—C107—C119—C118	56.1 (11)
C8—C7—C19—C14	−0.9 (8)	C108—C107—C119—C118	178.1 (8)
C4—O4—C1'—C2'	141.2 (6)	C104—O104—C11'—C16'	−88.5 (7)
C4—O4—C1'—C6'	−96.1 (6)	C104—O104—C11'—C12'	149.4 (5)
O4—C1'—C2'—C3'	178.0 (6)	O104—C11'—C12'—C13'	175.7 (6)
C6'—C1'—C2'—C3'	57.8 (9)	C16'—C11'—C12'—C13'	56.0 (9)
O4—C1'—C2'—C7'	−55.9 (9)	O104—C11'—C12'—C17'	−57.4 (8)
C6'—C1'—C2'—C7'	−176.2 (6)	C16'—C11'—C12'—C17'	−177.1 (7)
C1'—C2'—C3'—C4'	−57.9 (9)	C11'—C12'—C13'—C14'	−55.0 (10)
C7'—C2'—C3'—C4'	176.4 (7)	C17'—C12'—C13'—C14'	178.0 (8)
C2'—C3'—C4'—C5'	54.9 (11)	C12'—C13'—C14'—C15'	55.9 (11)
C3'—C4'—C5'—C6'	−51.8 (10)	C13'—C14'—C15'—C16'	−54.3 (10)
C3'—C4'—C5'—C10'	−177.1 (8)	C13'—C14'—C15'—C1T'	−176.8 (8)
C10'—C5'—C6'—C1'	176.5 (7)	C14'—C15'—C16'—C11'	54.4 (10)
C4'—C5'—C6'—C1'	52.4 (9)	C1T'—C15'—C16'—C11'	178.0 (8)
O4—C1'—C6'—C5'	−178.3 (6)	O104—C11'—C16'—C15'	−175.8 (6)
C2'—C1'—C6'—C5'	−56.0 (9)	C12'—C11'—C16'—C15'	−57.0 (9)
C1'—C2'—C7'—C9'	−63.9 (9)	C11'—C12'—C17'—C18'	177.5 (7)
C3'—C2'—C7'—C9'	60.4 (9)	C13'—C12'—C17'—C18'	−58.6 (10)
C1'—C2'—C7'—C8'	168.1 (7)	C11'—C12'—C17'—C19'	−58.8 (8)
C3'—C2'—C7'—C8'	−67.6 (9)	C13'—C12'—C17'—C19'	65.1 (9)

### Hydrogen-bond geometry (Å, °)

**Table:**

*D*—H···*A*	*D*—H	H···*A*	*D*···*A*	*D*—H···*A*
N1—H1N···O5^i^	0.88	2.19	3.015 (9)	157
O2—H2O···O3	0.84	2.46	2.877 (9)	111
N101—H11N···O105^ii^	0.88	2.15	2.977 (10)	155
O102—H12O···N101	0.84	2.31	2.761 (10)	114
O300—H30O···O2^i^	0.86	1.95	2.707 (12)	145
C3—H3···O1^ii^	1.00	2.26	3.222 (10)	162
C12—H12···O105^ii^	0.95	2.55	3.444 (9)	158
C103—H103···O101^i^	1.00	2.29	3.250 (8)	161

Symmetry codes: (i) *x*+1, *y*, *z*; (ii) *x*−1, *y*, *z*.
